# The FLORIVON flora survey in the Netherlands between 1902 and 1950

**DOI:** 10.3897/phytokeys.135.30069

**Published:** 2019-10-30

**Authors:** Laurens B. Sparrius, Joop van Heeswijk, Gerard M. Dirkse, Michiel J.J.M. Verhofstad

**Affiliations:** 1 FLORON Plant Conservation Netherlands, Toernooiveld 1, 6525 ED Nijmegen, The Netherlands FLORON Plant Conservation Netherlands Nijmegen Netherlands; 2 Naturalis Biodiversity Center, P.O. Box 9517, 2300 RA Leiden, The Netherlands Naturalis Biodiversity Center Leiden Netherlands

**Keywords:** Biodiversity, citizen science, digitization, mapping, taxonomy, vascular plants

## Abstract

In 1902, the nationwide citizen science project, known as FLORIVON, for mapping the flora of the Netherlands was launched, resulting in the publication of a complete flora atlas in 1980. Until 2004, the atlas dataset of the fieldwork between 1902 and 1950 had only been partly digitised and observations were aggregated and anonymised. Between 2001 and 2018, the dataset has been entirely digitised from the original field forms, including notes on non-native taxa. This paper presents key characteristics and figures of the dataset and provides an overview of the historical survey project, the digitisation process and subsequent validation of the data. The dataset is currently curated in the National Database Flora and Fauna and published in GBIF.

## A brief history of flora mapping in the Netherlands

In March 1902, the National Herbarium of the Netherlands (L) and the Dutch Botanical Society started a citizen science project – nowadays referred to as FLORIVON – to map the flora of the entire country of the Netherlands, led by J.W.C. Goethart and W.J. Jongmans (Goethart 1902). During the project, observations were noted by checking taxon names on a special recording form. A field survey was carried out for each map grid cell of 1.3 × 1.01 km.

Starting from the autumn of 1902 until 1907, small numbers of distribution maps were published on an irregular basis to show participants the progress of the work. From 1908 to 1923, only a few participants continued their work, mainly during the so-called *Unio* summer meetings of the Dutch Botanical Society (Smit and Verschoof 1980). In 1924, a new group of botanists, led by J.L. van Soest and J.G. Sloff, continued the mapping project (Verschoof 1978). Another group, led by W.C. de Leeuw, focused on mapping the changes in the flora after the construction of the Afsluitdijk, a dam that caused the Zuiderzee to transform from a salt water body into the a freshwater lake (Westhoff 1964).

In 1930, the IVON foundation (Institute for Vegetation Research in the Netherlands) was founded by J.W.C. Goethart and aimed to unite all botanists working on plant surveys. Between 1930 and 1939, many grid cells were surveyed and preliminary maps were compiled and published in several journals (e.g. Sloff 1935). During and after World War II, the survey project slowed down. Although the project never formally ended, 1950 could be considered as the final year of the field surveys.

It was only in 1980 that the data were compiled into an atlas of the flora of the Netherlands with maps on 5 × 5 km spatial resolution. The atlas was produced by J. Mennema and co-workers at the National Herbarium in Leiden (Mennema et al. 1980).

In 1988, FLORON was founded as a spin-off from the National Herbarium to continue the vascular plant surveys by volunteers and build a database by digitising distribution data of vascular plants. At first, the Atlas of the Flora of the Netherlands (published in 1980) was digitised to have quick access to historical distribution maps. Between 2001 and 2018, all original field forms, opportunistic observations on handwritten notes, letters, vegetation relevées and literature data were digitised by Joop van Heeswijk and compiled into the FLORIVON dataset which is described in this paper.

## Methods

### Sampling protocol

**Figure 1.**
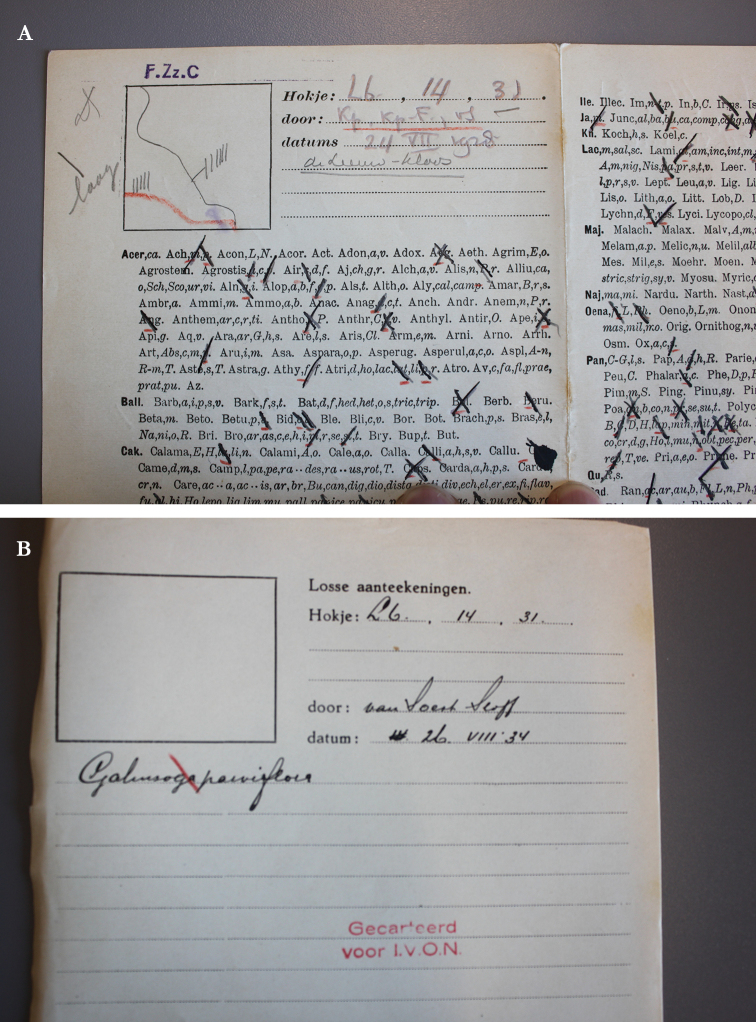
Samples of the FLORIVON survey forms: **A** field form showing a square with a drawing of the surveyed area, space for writing down grid square code (‘hokje’), location name, observer name(s) and date, followed by two pages of taxon abbreviations that surveyors had to cross out after observation **B** written note with header data containing the grid cell code (e.g. L6.12.31), observer’s name and the survey date. Stamps confirm that the data have been included in printed atlas volumes.

The basis of the survey scheme was a map of grid cells 1.3 × 1.01 km covering the Netherlands. Grid cells were assigned to participants by the project organisation. Each grid cell was then surveyed for several hours to one day aiming to make a complete list of all wild vascular plants occurring in the area. Survey data were recorded on field forms with abbreviations of scientific taxon names printed on them (Fig. 1). Nomenclature followed the second edition of the Prodromus Florae Batavae (Vuyck 1901). Additionally, miscellaneous observations, vegetation relevées and literature records (from 1832 until 1953) were submitted on special forms or in handwritten or typewritten letters. Most observations include the grid cell code, taxon name, date and up to 9 names of co-observers (Table 1). In total, 56,103 forms were digitised, of which 47,060 were field survey forms, 8,279 written notes and 764 vegetation relevées. The average number of taxa per form was 47. Most of the field forms contained higher numbers of taxa, while written notes usually reported only 1–5 taxa (Table 2).

**Table 1.** Top four observers per decade in the main survey period of FLORIVON with the number of surveys performed in brackets. In total, 572 people were mentioned as observers in the dataset.

1900–1909	1910–1919	1920–1929	1930–1939	1940–1949
L. Vuyck (2369)	L. Vuyck (1477)	A. Koopmans (867)	J. Sloff (6722)	J.L. van Soest (768)
J.W.C. Goethart (1293)	A.W. Kloos (577)	D. Koopmans-Forstmann (634)	J.L. van Soest (3448)	J. Sloff (715)
W.J. Jongmans (1123)	J. Sloff (407)	J.L. van Soest (254)	T. Weevers (2187)	G. Sissingh (417)
M.J. Blijdenstein (998)	D. Lako (284)	A.W. Kloos (189)	Joh. Jansen (1756)	V. Westhoff (364)

**Table 2.** Number of taxa recorded per form in the FLORIVON dataset.

Number of taxa mentioned on a survey form	Number of survey forms
1–5	11089
6–11	7495
12–25	8212
25–50	8516
50–00	13310
100–378	8080

### Data processing and quality control

Survey forms were digitised using Turboveg (Hennekens and Schaminée 2001), a computer programme usually used for handling phytosociological relevées, with customised species dictionaries matching the taxonomy and nomenclature of the field survey forms. All additional written information on the forms, including additional taxon names of, for example, non-native taxa, additional survey dates and remarks were temporarily included in the Turboveg header record and extracted afterwards.

Taxon names were mapped to current names using a translation table between the Prodromus Flora Batavae (Vuyck 1901) and a more recent checklist of vascular plants in the Netherlands (Groen et al. 1999). The original taxon name or its abbreviation is kept in the database. Grid cells codes were translated to geographical coordinates. Observer names were mapped to existing observer identifiers in the National Database Flora and Fauna.

Records without an observation date were assigned to the entire survey period of 1902–1950. Records without a valid taxon name or missing grid cell codes were omitted from the final dataset. Records with locations entirely outside the country or in the sea were also omitted. A total of 5,530 records were cleaned. The number of digitised observations after this first data cleaning step was 2,638,919.

Validation of the digitised observations was performed with an automated procedure which involved trying to find a match for each observation in a dataset, based on printed volumes of the Atlas of the Flora of the Netherlands (Mennema et al. 1980) and other digitised literature and collection records in the National Database Flora and Fauna, which had been validated in the past.

In the FLORIVON dataset, 142,838 observations did not match validated data sources and were considered for a manual review. Of the remaining unmatched observations, 110,889 records of common taxa were validated, i.e. taxa occurring in 30% or more of the 5 × 5 km grid squares in the Netherlands. A total of 2,415 records of less common taxa were validated if they were present in neighbouring grid cells. Further unmatched records, rare taxa, were validated by Gerard Dirkse by plotting them on a map for visual interpretation (17,427 observations). These observations were validated if they matched the geographical pattern of all other valid observations of the taxon. Herbarium specimens and publications mentioning an observation were also taken into account during validation. In the validation process, 12,107 out of 142,838 records were deleted (ca. 3%).

The validated dataset was added to the NDFF Verspreidingsatlas (http://www.verspreidingsatlas.nl), which is the platform FLORON uses to curate datasets. Simultaneously, the dataset was published through the GBIF Integrated Publishing Toolkit (IPT).

### Personnel

Joop van Heeswijk performed the digitisation between 2001 and 2018 as voluntary work. Laurens Sparrius performed the validation of the dataset. Gerard Dirkse assisted with the validation of non-native and doubtful taxa. Naturalis Biodiversity Center (Leiden) is hosting the physical archive with field forms and notes.

## Dataset

### GBIF Dataset description

The dataset is curated on the NDFF Verspreidingsatlas data platform and will be updated on GBIF annually if any changes are made. Included Darwin Core terms are: occurrenceID, type, language, licenserightsHolder, accessRights, references, datasetName, basisOfRecord, eventDate, decimalLatitude, decimalLongitude, geodeticDatum, coordinateUncertaintyInMeters, scientificName, kingdom, taxonRank, scientificNameAuthorship.

Excluded information: Complete observer biographies, source type (field list, publication, specimen, vegetation relevée), location names and remarks were not included in the published dataset, but can be found in the source (curation) database, which can be accessed with the link below. This information was excluded due to privacy reasons or because it was deemed irrelevant.

**Object name**: FLORIVON

**Format name**: Darwin Core Archive format

**Format version**: 1.0

**Character encoding**: UTF-8

**Language**: English

**Licence**: http://creativecommons.org/licenses/by-nc/4.0/legalcode

**First publication date**: 2019/09/01

**Distribution**: http://www.verspreidingsatlas.nl:8080/ipt

**DOI**: https://doi.org/10.15468/ke2ody

**Curation website**: https://www.verspreidingsatlas.nl/waarnemingen

**Number of records**: 2,626,773

### Taxonomic coverage

The dataset only includes taxa of vascular plants (Kingdom Plantae: clade Tracheophyta). Most of the taxa are native to the Netherlands. Occasionally, non-native taxa were recorded. Nomenclature follows the last edition of the Flora of the Netherlands (van der Meijden 2005). Non-native taxa not listed in this Flora follow The Plant List (The Plant List 2013).

The dataset contains distribution data of 2502 taxa at species or intraspecific level divided over 138 plant families. The plant families with the most observations in the dataset belong to the Asteraceae and Poaceae (Table 3).

Some taxa in FLORIVON are currently accepted as lumped taxa, which makes it impossible to compare taxon distributions for certain taxa (Table 4).

**Table 3.** Top-25 of 137 plant families in the FLORIVON dataset.

Plant family	Number and percentage of observations
Asteraceae	326856 (12.4%)
Poaceae	292948 (11.1%)
Fabaceae	142223 (5.4%)
Rosaceae	124470 (4.7%)
Caryophyllaceae	108542 (4.1%)
Lamiaceae	108048 (4.1%)
Apiaceae	103661 (3.9%)
Plantaginaceae	100695 (3.8%)
Brassicaceae	91438 (3.4%)
Polygonaceae	75203 (2.8%)
Cyperaceae	71567 (2.7%)
Ranunculaceae	63497 (2.4%)
Juncaceae	50209 (1.9%)
Primulaceae	42433 (1.6%)
Amaranthaceae	33551 (1.2%)
Boraginaceae	33050 (1.2%)
Rubiaceae	32086 (1.2%)
Salicaceae	30801 (1.1%)
Ericaceae	29673 (1.1%)
Caprifoliaceae	28348 (1%)
Betulaceae	26600 (1%)
Violaceae	24663 (0.9%)
Onagraceae	23652 (0.9%)
Urticaceae	22418 (0.8%)
Orchidaceae	21735 (0.8%)

**Table 4.** Taxa in the Prodromus Florae Batavae and FLORIVON that are now considered lumped taxa.

Scientific names of combined taxa	Number of observations
Myosotis laxa subsp. cespitosa / scorpioides	14792
*Festuca rubra* / *arenaria*	14738
*Agrostis stolonifera* / *gigantea*	8930
*Betula pendula* / *pubescens*	7961
*Juncus bufonius* / *ambiguus*	6991
*Ranunculus aquatilis* / *peltatus*	5881
*Arenaria leptoclados* / *serpyllifolia*	5660
*Polypodium vulgare* / *interjectum*	5283
*Dryopteris carthusiana* / *dilatata*	4700
*Bolboschoenus maritimus* / *laticarpus*	3979
*Thymus pulegioides* / *serpyllum*	3326
*Atriplex prostrata* / *longipes*	2781
*Nasturtium microphyllum* / *officinale*	2279
*Aphanes arvensis* / *australis*	1955
*Potamogeton pusillus* / *berchtoldii*	1649
*Agrostis canina* / *vinealis*	1496
*Scrophularia auriculata* / *umbrosa*	1375
*Salicornia europaea* / *procumbens*	1333
*Veronica anagallis-aquatica* / *catenata*	1319
*Viola reichenbachiana* / *riviniana*	1296
*Malva neglecta* / *pusilla*	1263
*Elytrigia atherica* / *maritima*	785
*Festuca brevipila* / *lemanii*	428
*Ranunculus aquatilis* / *baudotii*	418
*Glyceria notata* / *declinata*	226
*Aster lanceolatus* / *ontarionis*	188
*Galeopsis ladanum* / *angustifolia*	121
*Trifolium campestre* / *dubium*	103
*Cerastium pumilum* / *glutinosum*	35

### Temporal coverage

**Figure 2.**
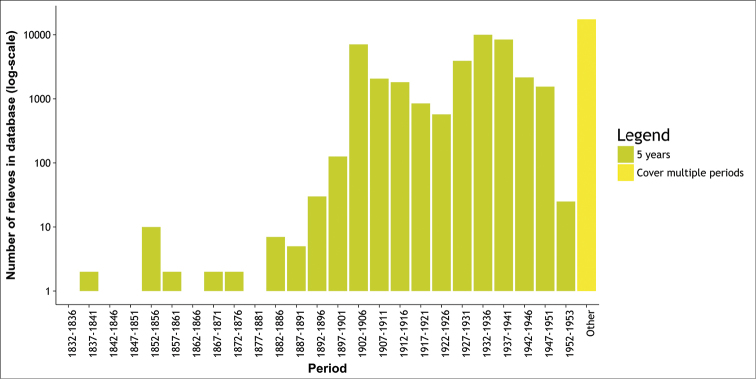
Number of surveys per 5 year period during the course of FLORIVON project.

The dataset contains observations and literature data from 1832 to 1953. Most of the data were collected between 1902 and 1950 as part of the FLORIVON citizen science project (Fig. 2).

### Spatial coverage

The dataset covers the entire country of the Netherlands as it was in the period 1902–1950. At that time, the southern part of the province of Flevoland did not yet exist (Hoeksema 2007). Additionally, minor changes were made to the border with Germany and Belgium after World War II (Wijchgel 2008).

**Figure 3.**
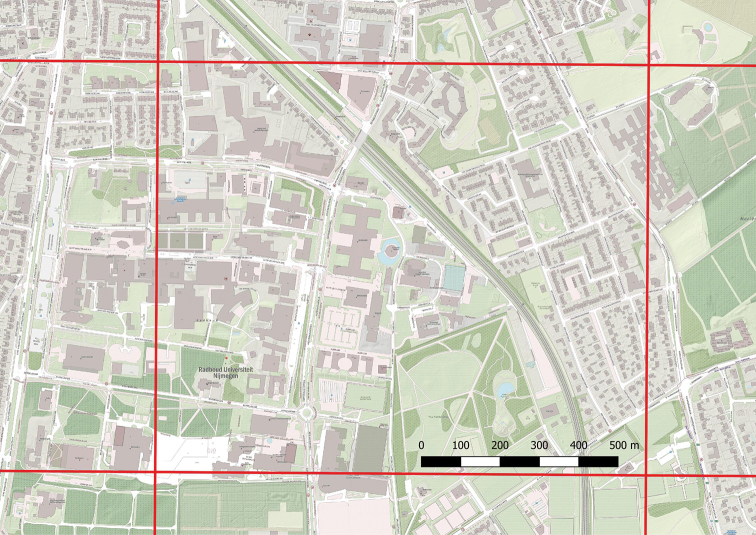
Example of a FLORIVON grid cell of 1.3 × 1.01 km, the smallest spatial unit in which data were collected. Map: OpenStreetMap.

Survey data were collected in small grid cells of 1.3 × 1.01 km (*kwartierhok*) (Fig. 3), 16 of which can be combined into a larger grid cell of 5.0 × 4.167 km (*uurhok*), which is used on some forms. The grid system was created in 1902 by the botanical community itself because, until 1920, a km grid was lacking on the topographical military maps. These grid cells differ from the currently used grid, in which the smallest grid cells are 1 × 1 km and follow the Dutch National Coordinate Reference System (ESPG: 28992).

**Figure 4.**
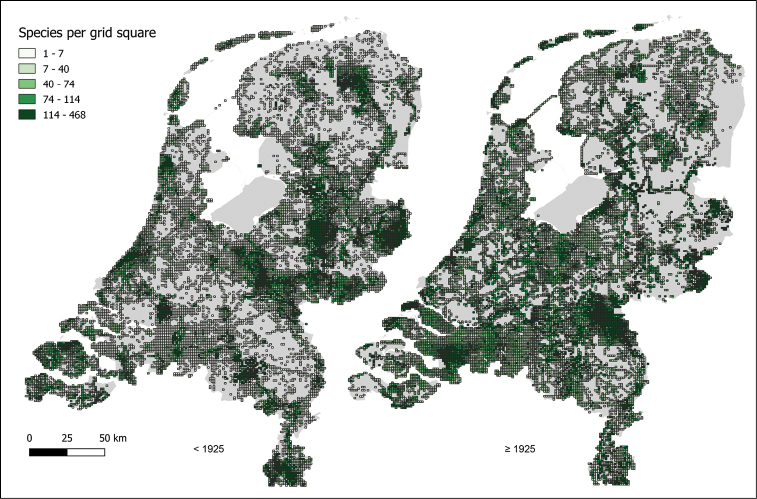
Maps of the Netherlands showing the number of taxa recorded per grid cell before and after 1925 in the context of the FLORIVON project.

The periods before and after 1925 show different patterns of survey intensity, which should be taken into account when using the data for further analysis (Fig. 4).
